# Transforming grayscale MRI images to color utilizing generative artificial intelligence to better understand multiple sclerosis

**DOI:** 10.1177/11795735241310138

**Published:** 2025-01-05

**Authors:** Darin T. Okuda, Christine Lebrun-Frénay

**Affiliations:** 1 Department of Neurology, Neuroinnovation Program, Multiple Sclerosis & Neuroimmunology Imaging Program, 12334 The University of Texas Southwestern Medical Center, Dallas, TX, USA; 2 Peter O’Donnell Jr. Brain Institute, 12334 The University of Texas Southwestern Medical Center, Dallas, TX, USA; 3 CRCSEP, 56814 Université Nice Cote d’Azur, Nice, France

**Keywords:** Multiple sclerosis, MRI, color, artificial intelligence

## Abstract

Multiple sclerosis (MS) falls within the spectrum of central nervous system (CNS) demyelinating diseases that may lead to permanent neurological disability. Fundamental to the diagnosis and clinical surveillance is magnetic resonance imaging (MRI) that allows for the identification of T2-hyperintensities associated with autoimmune injury that demonstrate distinct spatial distribution patterns. Here, we describe the clinical experience of a 31-year-old, right-handed, White man seen in consultation at The University of Texas Southwestern Medical Center in Dallas, Texas, following complaints of headaches that began after head trauma related to military service. Imaging data spanning over 10 years are provided. All MRI data are currently presented in black and white with grayscale values within voxels associated with a single variable, intensity. We transformed these grayscale values into color using generative artificial intelligence (AI). As color allows for the inclusion of three variables: hue, lightness (intensity), and saturation, we hypothesized that additional details may be learned beyond those currently provided with the existing conventional approach of grayscale interpretation. We identified differences in lesion colors that remained consistent from the two MRI timepoints studied. In addition, quantitative R1, R2, and proton density voxel values appeared consistent with the color scheme generated by the AI system. With advancing AI methods and capabilities along with the additional data that color provides in comparison to grayscale, new insights into the biology of disease may be possible. Modifying what we measure in people with chronic conditions and how we present the data may be of greater value than conventional approaches typically used in the study, education, and care of people with MS and other neurological conditions.

## Plain language summary

Multiple sclerosis (MS) is a chronic autoimmune condition involving the brain and spinal cord that may result in permanent neurological disability. Magnetic resonance imaging (MRI) studies are used both in the diagnosis and surveillance of people with MS. Current MRI data appear black and white in appearance and grayscale values within voxels comprise the images that are seen. However, grayscale values are associated with a single variable, intensity. On the other hand, color is associated with three variables: hue, lightness (intensity), and saturation. Overall, grayscale images are less dynamic and, more importantly, they may exclude existing details. We hypothesized that the transformation from grayscale MRI data to color may provide additional information related to lesions resulting from MS. We were able to identify color differences in lesions when evaluating MRI images from a single subject. These color changes appeared to remain consistent over the course of 10 years. Quantitative MRI values also suggested a relationship between degree of brain injury within lesions by color. With advancing artificial intelligence methods and capabilities along with the addition of color information, more could be learned about the biology of MS. The information provided here suggests that the use of color may have value in neuroimaging. Adjusting what we measure for those within chronic conditions and how the information is presented may be of greater value than current practices and may enhance the study, education, and delivery of care for those with chronic neurological conditions.

## Introduction

Multiple sclerosis (MS) falls within the spectrum of central nervous system (CNS) demyelinating disease and is associated with both acute and progressive clinical courses,^1,2^ resulting in varying degrees of neurological impairment.^3,4^ Individuals asymptomatic for MS may also be recognized with typical T2-hyperintensities within the CNS found incidentally when an MRI study is performed for other reasons.^5-7^ Observed intra-axial and intramedullary high-signal anomalies representing multi-focal regions of autoimmune related inflammation due to misrecognition of normal tissue by the immune system on magnetic resonance imaging (MRI) studies are critical to the diagnosis of MS.^1^ Such MRI findings are also meaningful for clinical surveillance in the context of disease modifying therapy treatment or more conservative approaches to care.

High-signal anomalies associated with MS are easily recognized on MRI due to the remarkable sensitivity of the technology. Since inception, images have been provided in grayscale, displaying a spectrum of shades of gray that represent signal intensities from normal anatomical structures along with the pathological effects of disease. Observed contrast differences in signal intensities enable differentiation between different tissue types but also may indicate the advancement of disease. The transformation of grayscale MRI images to color may provide further clarity in rapidly identifying changes associated with disease advancement. Reviewing full colorized image data may also readily provide additional details that are less apparent in grayscale images.

Both community and academic neurologists frequently order MRI studies to better understand symptoms described by patients. When results are discussed, the images are routinely displayed and explained to the patient. To many, the provided images appear intimidating, not easily intuitive, and are difficult to understand. The application of colorized MRI data may serve as an aesthetic improvement, creating an experience that can be more engaging and visually appealing. Data in this format may also be of greater benefit to clinicians that have less experience in reviewing MRI data.

In this report, we describe the clinical experience of a person with MS and transform grayscale MRI data to color using generative artificial intelligence (AI) to determine the potential value of this technique in better understanding the imaging features of MS. A comparison of the observed T2-hyperintensities in color to acquired quantitative MRI data are also provided to assess the effectiveness of the colorization process.

## Methods

### Clinical experience description

**Figure 1.**
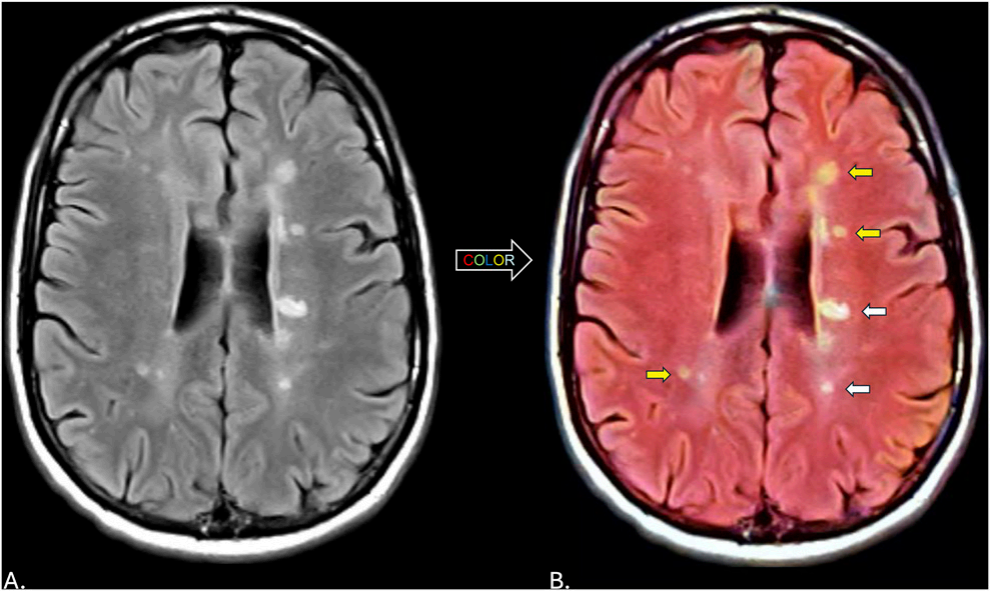
(A) Grayscale axial 3.0 Tesla fluid attenuated inversion recovery image of the brain from January 2014 demonstrating T2-hyperintense foci with spatial distribution characteristic for multiple sclerosis. (B) Transformation of the grayscale image to color using generative artificial intelligence. Note the color differences in select lesions highlighted with some appearing more yellow (yellow arrows) and others white (white arrows).

A 31-year-old, right-handed, White man was seen in consultation at The University of Texas Southwestern Medical Center in Dallas, Texas, following complaints of headaches that began after head trauma related to military service. His headache was accompanied with nausea and vomiting, persisting for a few days. His history was negative for prior events of vision loss or focal motor/sensory complaints. A comprehensive neurological examination was performed and found to be normal. A brain MRI study was performed that revealed features highly suggestive of inflammatory CNS demyelination based on the size, number, morphology and spatial distribution of T2-hyperintensities (Figure 1). A dedicated MRI study of the cervical spinal cord was unremarkable for intramedullary lesions. Cerebrospinal fluid analysis revealed the presence of >2 unique oligoclonal bands.

The patient opted to participate in the first randomized clinical trial aimed at preventing or delaying the onset of a first clinical event related to MS.^8^ Within the study, he was randomized to the active treatment arm, dimethyl fumarate (Tecfidera®) and remained symptom free. However, following the 96-week study, he developed a new spinal cord lesion that was associated with long tract motor and sensory symptoms despite full compliance with dimethyl fumarate. Given the evolution of symptoms resulting from inflammatory CNS demyelination and prior evidence of new lesion development on MRI, he fulfilled the diagnosis of relapsing-remitting MS. Following acute management of symptoms that resulted in full recovery, his disease modifying therapy was switched to ocrelizumab (Ocrevus®).

### Artificial intelligence colorization technique

A deep learning network, trained on a diverse image dataset was created. The preprocessing pipeline converted the input of red, green, and blue (RGB) images into grayscale while maintaining the RGB channel structure. During training, the network learned the mapping between grayscale and full-color version, with the loss function calculated by comparing the network output against the original RGB values from the training images. Grayscale Digital Imaging and Communications in Medicine files were then converted into a feature representation via a convolutional neural network. Simultaneously, an accompanying text prompt was transformed into a feature vector using natural language processing techniques. The image and text feature vectors were subsequently entered into a deep generative network, which was trained for function approximation from the MRI grayscale image and text features to generate a colorized version of the image.

### Magnetic resonance imaging data

In January 2014, a 3.0 Tesla (T) brain MRI was performed containing a 2-dimensional (2D) fluid attenuated inversion recovery (FLAIR) sequence, gadolinium-enhanced 3-dimensional (3D) T1-weighted gradient echo sequence, and a 2D T2-weighted sequence. For comparison, images acquired from a repeat 3.0 T MRI performed more than 10 years later in June 2024 were used. The following sequences were acquired: i) 3D T2-weighted sequence, ii) 3D FLAIR sequence, and iii) a non-enhanced 3D T1-weighted gradient echo sequence. Quantitative 3D data using an interleaved Look-Locker acquisition sequence with T2 preparation pulse was also acquired to obtain quantitative T1, T1, and proton density values from regions of interest (SyntheticMR, Linköping, Sweden).

## Results

**Figure 2.**
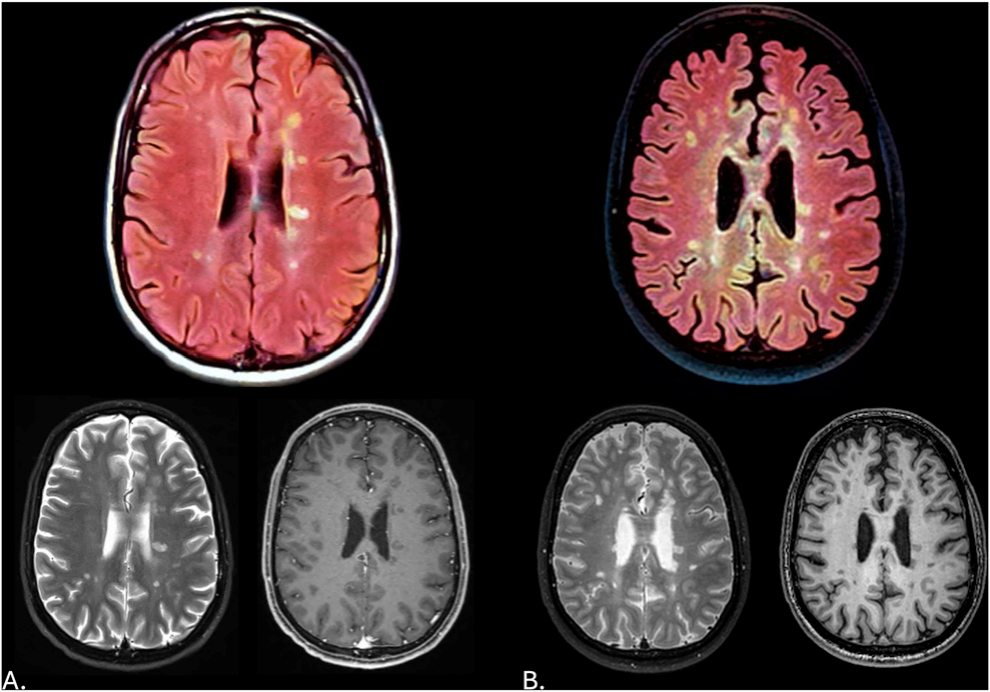
(A) Color transformed axial 3.0 Tesla (T) fluid attenuated inversion recovery (FLAIR) image of the brain with corresponding T2-weighted and post-contrast T1-weighted images at the same level from January 2014. (B) Color transformed axial 3.0 T FLAIR image with corresponding T2-weighted and T1-weighted images at the same level from June 2024. Note the changes in lesion number and size along with the generalized reduction in brain volume.

**Figure 3.**
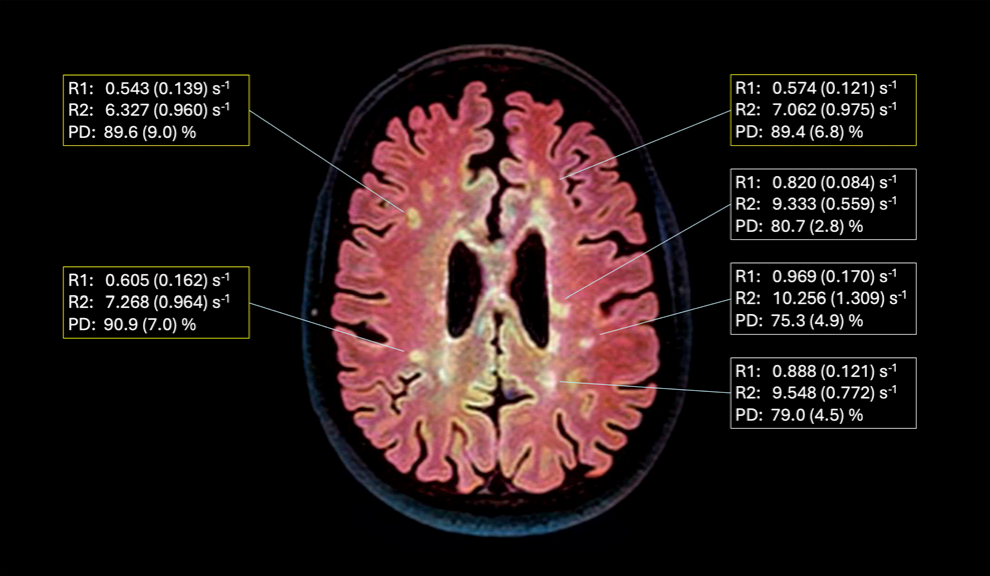
Color transformed axial 3.0 Tesla fluid attenuated inversion recovery image of the brain from June 2024 with corresponding quantitative R1, R2, and proton density mean and standard deviation values from selected lesions. Note the differences in quantitative values when comparing lesions appearing more yellow than white.

The transformation of grayscale MRI FLAIR images to color via generative AI revealed heterogeneity in the T2-hyperintensities present with some of the lesions having a more yellow appearance and others appearing more white in color (Figure 1). Data from other accompanying sequences (T1-weighted and T2-weighted sequences) at either time point did not appear to provide a clear explanation for the observed variations in lesion color (Figure 2). Over a time period of 10 years, and despite treatment with a disease modifying therapy, radiological advancement was observed with the interval development of new T2-hyperintense lesions present along with a reduction in total brain volume. Previously identified lesions from the first MRI study (Figure 1) with distinct colors appeared to have a similar appearance in 2014, although enlargement of some of the existing lesions were present. For those lesions having a more yellow appearance, lower quantitative R1 (longitudinal relaxation rate represented by 1/T1 relaxation time) and R2 (transverse relaxation rate represented by 1/T2 relaxation time) and higher proton density (measure of the concentration of hydrogen protons) values were identified (Figure 3).

## Discussion

The first photograph in color was produced by James Clerk Maxwell in 1861, a Scottish physicist, using red, blue, and yellow filters. A colored image of a tartan ribbon was formed after combining the individual images in red, green, and blue-violet into one color composite.^9^ Beyond pursuing his interest in trichromatic theory, more simply, the colored image provided greater realism and detail when compared to black and white.

In this report, we demonstrated the potential value of transforming grayscale MRI images into color, visually enhancing the anatomical data and possibly providing more information that may inform on the impact of disease related to MS within the CNS. Colorizing MRI data appears to offer more intuitive data to the observer. In addition, the application of generative AI color rendering techniques may enable better appreciation of findings not apparent in grayscale. When correlated with quantitative metrics, the more yellow-colored lesions appeared to have lower R1 and R2 values and higher proton density values when compared to lesions that were white. Higher T1 relaxation time values are observed within persistent T1 “black holes” on MRI, given the reduction in tissue integrity.^10^ Collectively, our observation of higher R1 and R2 findings and higher proton density values, representing a higher water content, suggests that variations in color may better allow for the appreciation of greater demyelination and axonal compromise within lesions. Lesions related to MS are well known to evolve in shape, size, and location on MRI^11-13^ and the degree of tissue compromise within the selected lesions studied here also appears to vary widely. As generative AI methodology continues to improve at a rapid pace and given the increasing prevalence of use within healthcare, advancements in the near immediate analysis of existing clinical MRI data may be possible.

Grayscale images are ideal in demonstrating contrast, enabling the differentiation of tissue types or complexities within lesions. However, grayscale values within voxels on MRI are only associated with a single variable, intensity. The visual evaluation of data in grayscale has been shown to impact our perceptions of size estimation as well as perceived changes.^14-16^ These limitations in our visual system is the result of Mach bands where a perceived increase in contrast is observed at boundary regions of contrasting shades^17^ and result from visual processing of lateral inhibitory optic neurons that are involved in enhancing contrast and edge detection.^18^ On the other hand, color allows for the inclusion of three variables: hue, lightness (intensity), and saturation.^19^ As a result, the visual system processes hue and saturation data, reducing the Mach band effect. Intuitively, these additional variables may change the overall experience by viewers, improving the appreciation of imaging data in color and potentially offering additional details not readily apparent when viewing conventional grayscale data. The use of color in comparison to use of black and white in multimedia has also been associated with better attention and memory performance^20^ and object recognition.^21^

Previous work aimed at transforming medical imaging data into color has been limited. One of the earliest attempts aimed to transform MRI data to better study the female pelvis.^22^ Color-coded MRI multiparametric maps have also been studied for the diagnosis of prostate cancer.^23^ The application of automated color-coding on longitudinal MRI imaging data has been studied in both MS^24,25^ and brain metastases^26^ to more easily recognize interval radiological changes. In these efforts, color is applied to more clearly emphasize expansion or reduction of the region of interest. Here, an example of the full transformation from grayscale to color is provided through generative AI. Although many AI platforms exist to accomplish such tasks, each is unique, yielding a spectrum of outputs, with the vast majority lacking biological meaning. Being able to generate the ideal hues, intensities, and saturation that hold scientific significance is the fundamental goal.

## Conclusion

The application of color in neuroimaging may provide greater contextual clarity in comparison to current grayscale images for those without reduced sensitivity to red, green, and blue light. With advancing AI methods and capabilities along with the additional data that color provides in comparison to grayscale, perhaps new insights into the biology of disease may be recognized. More studies are needed not only to clarify the most ideal augmented intelligence methodologies for color transformation but also our understanding of how the software systems accomplish this task. Modifying what we measure in people with chronic conditions and how we present the data may be of greater value than conventional approaches typically used in the study, education, and care of people with MS and other neurological conditions.
